# Study protocol: a randomised controlled trial of supervised resistance training versus aerobic training in Sri Lankan adults with type 2 diabetes mellitus: SL-DART study

**DOI:** 10.1186/s12889-018-5069-6

**Published:** 2018-01-24

**Authors:** C. Ranasinghe, A. P. Hills, G. R. Constantine, G. Finlayson, P. Katulanda, N. A. King

**Affiliations:** 1 School of Exercise and Nutrition Sciences & Institute of Health and Biomedical Innovation Queensland University of Technology, Brisbane, Australia; 2 Department of Allied Health Sciences, Faculty of Medicine, University of Colombo, Sri Lanka. No 25, Kynsey road, Colombo, 08 Sri Lanka; 3 College of Health and Medicine, University of Tasmania, Launceston, Australia; 4 Department of Clinical Medicine, Faculty of Medicine, University of Colombo, Colombo, Sri Lanka; 5 School of Psychology, Faculty of Medicine and Health University of Leeds, Leeds, UK

**Keywords:** Exercise, Physical activity, Progressive exercise, Resistance, Aerobic, Type 2 diabetes mellitus, Diabetes, Randomized controlled trial, Adherence

## Abstract

**Background:**

The prevalence of type 2 diabetes mellitus (T2DM) and cardiovascular diseases (CVD) is rising globally. T2DM is particularly problematic in South Asia with an estimated 10–15% of Sri Lankans diagnosed with the disease. Exercise is known to improve blood glucose, lipid profiles, blood pressure and adiposity, key goals in the management of T2DM. However, much of the evidence to date has been gained from white Caucasians who have a different body composition and disease profile compared to South Asians. Similarly, the recreational exercise culture is new to Sri Lankans and the effects of exercise on T2DM has not been studied in this population.

**Methods:**

The Sri Lanka Diabetes Aerobic and Resistance Training (SL-DART) Study will be comprised of 2 components. Component 1 is a 12-week randomized controlled trial (RCT) to compare the effects of a supervised progressive resistance exercise program (RT) and aerobic exercise program (AT) with standard treatment/control (CN). Sedentary Sri Lankan adults with T2DM (aged 35–65 years) and with no contraindications to exercise will be randomized into one of 3 groups (AT, RT, CN). Exercise sessions will be conducted 2 days/week for 3 months. Baseline and post-intervention biochemical (glycemic control, lipid and liver profiles, inflammatory markers), anthropometric (height, weight, body circumferences), body composition, physical fitness, food preference (liking and wanting food) and quality of life parameters will be measured and compared between groups. Component 2 will be a qualitative study conducted immediately post-intervention via in-depth interviews to assess the barriers and facilitators for adherence to each exercise program.

**Discussion:**

SL-DART Study represents one of the first adequately powered methodologically sound RCTs conducted in South Asia to assess the effects of resistance and aerobic exercise in participants with T2DM. Triangulation of quantitative and qualitative outcomes will enable the design of a culturally appropriate therapeutic physical activity intervention for Sri Lankans with T2DM, and the initiation of a professionally driven and specialized clinical exercise prescription service.

**Trial registration:**

Sri Lanka Clinical Trials Registry; SLCTR/2016/017. Date registered 17.06.2016. Universal trial number U1111–1181-7561.

## Background

Type 2 diabetes mellitus (T2DM) and cardiovascular diseases (CVD) are major contributors to rising global mortality [1–3]. South Asia is home to almost one-fifth of the world’s population [4], a sub-population more prone to T2DM compared to other ethnicities [5]. In addition, an estimated 25 million people of South Asian origin (India, Pakistan, Bangladesh, Nepal, and Sri Lanka) [4] are living outside the region and similar prevalence rates have been reported in migrants in the USA, Canada, and various European countries [5]. There is a high prevalence of T2DM (10–15%) in South Asian Sri Lankans [6] who are at a greater risk of CVD [7] and develop T2DM at a younger age with increased incidence of complications (retinopathy, nephropathy, and coronary artery and cerebrovascular disease) than white Caucasians [5].

A number of explanations have been proposed to explain these ethnic differences, most commonly differences in body composition. For example, compared to white Caucasians, South Asians have increased abdominal adiposity/central obesity leading to insulin resistance (IR), T2DM and CVD [5]. This phenomenon can be explained in ‘adipose tissue compartment overflow hypothesis’ [8], stating the superficial subcutaneous tissue is less developed in South Asians, resulting in early expansion of the visceral and deep subcutaneous compartments [5, 9]. In addition the ‘thrifty genotype hypothesis’ states some people (possibly South Asians) can possess genes facilitating increased fat storage during food abundance for survival during later famine [10].

Sri Lanka is also experiencing a socio-economic or nutrition transition, a change from a more active agricultural lifestyle to a more sedentary lifestyle due to industrialization and urbanization and resultant overweight and obesity [6]. Historically, structured recreational exercise has not been a part of the culture in Sri Lanka [11] and it is uncommon to see recreational exercise groups, gymnasia and professionally trained staff. Poor food choices are also contributing to less than desirable health outcomes. For example, only 3–5% of Sri Lankan adults consume the recommended 5 portions of fruits and vegetables per day and diets are characterized by higher portions of rice and less protein (mainly by rice and pulses), almost half that of US adults [12]. Prevention and management of an estimated 1–1.5 million people with pre-diabetes and diabetes is causing an escalating burden to the Sri Lankan economy and society. There is a need to develop effective management strategies to prevent a worsening of the problem.

Exercise is known to improve blood glucose, lipid profiles, blood pressure [13] and visceral adiposity [14], key goals of diabetes management, with food intake and quality of life also considered as important factors. Combined aerobic exercise training (AT) and resistance exercise training (RT) interventions have been widely utilized in white Caucasians populations [15]with benefits also reported for either exercise modality when used individually [16, 17]. It has been suggested that South Asians could benefit more from RT due to their relatively higher adiposity and lower lean body mass compared to Caucasians [18]. Currently, no Sri Lankan studies have documented and very few South Asian studies have compared the effects of AT plus RT on people with T2DM. Secondly, low compliance and adherence is a common drawback in exercise interventions with information on the qualitative factors associated with non-adherence to exercise often overlooked [19]. Finally, relatively few South Asian studies have addressed food intake and the effects of AT and RT on food preference [20].

### Aims of the study

The primary aim of the Sri Lanka Diabetes Aerobic and Resistance Training (SL-DART) Study is to investigate the effect of two exercise modalities in Sri Lankan adults (aged 35–65 years) diagnosed with T2DM. A 12-week randomized controlled trial (RCT) will compare AT, RT and usual care on the primary outcome measure, glycemic control (HbA1c). Secondary outcome measures include percentage body fat, blood lipid and liver profiles, inflammatory markers, muscle strength, cardiovascular endurance, blood pressure, quality of life attributes and food preferences. A secondary aim is the qualitative assessment of barriers/facilitators to compliance and adherence to different modes of exercise via in-depth interviews.

To the best of our knowledge, a study of this nature has not been conducted in Sri Lanka or in other parts of South Asia. In a culturally diverse setting like Sri Lanka, the development of a feasible exercise intervention for T2DM would therefore be very novel. The experiences of SL-DART Study will enable the development of a culturally acceptable clinical exercise physiology service to people with T2DM.

## Methods

### Study design

**Fig. 1:**
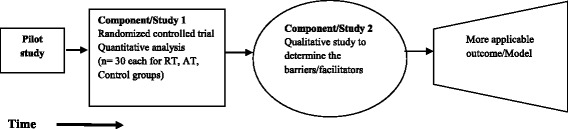
Research plan

**Fig. 2:**
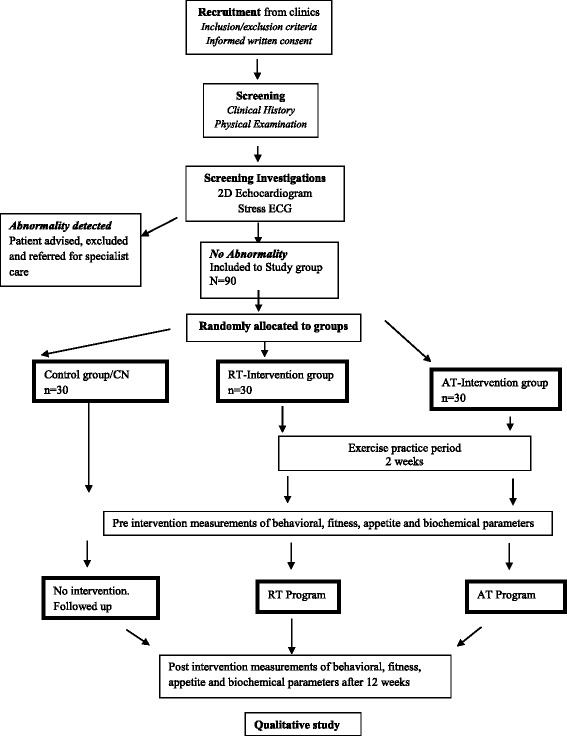
Flow chart of sampling, recruitment and progression of RCT/Study 1

- Pilot study
- Component 1: A 12-week RCT will study the effects of a supervised RT and AT program on behavioral, anthropometric, physical fitness, appetite and biochemical parameters in sedentary Sri Lankan adults with T2DM (aged 35–65 years) with no contraindications to exercise. Outcomes will be compared between exercise programs and a control group who receive standard care.
- Component 2: A qualitative study will be undertaken immediately after the intervention to assess the barriers and facilitators for adherence to each program (refer to Figs. 1 and 2).

### Settings and study population

The study will be conducted in the Faculty of Medicine, University of Colombo, Sri Lanka with training carried out at the University’s Strength Training Gymnasium. The study population will be recruited from patients attending the medical and diabetic clinics of the National Hospital Sri Lanka (NHSL) and private sector endocrine clinics in Colombo after prior approval from the consultant physician in-charge.

### Preliminary study/pilot study

A 4-week pilot study was conducted to pretest the main study design methodology. Nine (*N* = 9) participants were recruited into each group (*n* = 3, Male: Female AT 1:2, RT 1:2, CN 2:1) and all completed the intervention. Main outcome of the pilot study was the change in HbA1c.The baseline values for HbA1c (Mean, SD) were 8.0(1.4)/7.7 (1.7)/8.3%(1.7) and post intervention values were 7.8 (1.2)/ 7.4 (1.1)/ 8.2%(1.7) for AT, RT and CN respectively. Challenges were identified in the difficulty of recruitment, including excessive time taken. A trained pre-intern medical doctor was recruited for initial participant contact. Participant response and tolerance to the intervention helped determine the duration of the practice period (4 sessions) necessary to teach the technical skills. The feasibility of the exercise protocol was improved by confirming the frequency of participant attendance (2 sessions/week), initial intensity and progression of exercises, testing the safety and first aid protocols and specific time points for measuring intervention outcomes. Technical and logistical constraints associated with coordination between laboratories and institutions were identified and improved. For example, access to the gymnasium and the investigation laboratories was improved by providing free car parking and transport facilities when needed. The availability of the principal investigator and exercise administrators at all times resulted in increased retention of participants. Pre-testing of all questionnaires was also undertaken.

### Process evaluation

A number of strategies for documenting the implementation of the intervention were developed with the pilot study. Standard Operating Procedures (SOPs) were developed for participant recruitment, randomization, screening, conducting pre- and post-intervention measurements and implementation of the exercise intervention. Dose of exercise to be delivered and its progression, reach for the participants (logs of participant attendance, participant experience about the intervention) were included in the information booklet. The intervention fidelity was separately assessed. Before the start of the study, the methods by which they would be documented, who would be responsible for completing the documentation, and procedures for data entry and evaluation was decided and the investigators and exercise supervisors were trained accordingly.

### Eligibility criteria

Inclusion criteria of participants include:

1. Non-cognitively impaired Sri Lankan males and females (non-pregnant and not planning to fall pregnant), aged 35–65 years, and diagnosed with T2DM within the last 10 years.
2. Not participated in any exercise program within the previous 6 months.

Participants will be excluded if blood HbA1c levels < 6.5% or > 11.5%, on insulin or thiazolidinedione therapy/other than standard oral hypoglycemic agents, advanced diabetes induced end-stage organ damage and history of significant cardiovascular (ischemic heart disease etc.), respiratory (asthma, chronic obstructive pulmonary disease etc.), musculoskeletal diseases. To maintain accuracy, the patient informant data will be cross-checked with the patient health records in the clinic (e.g. HbA1c levels, duration of diabetes, drug history etc.) by the principal investigator who is an experienced medical doctor.

### Informed consent

Written informed consent will be obtained from all participants prior to their entry into the study.

### Sample size

Ninety (*n* = 90) participants will be recruited from a roll-in method and allocated to RT group (*n* = 30), AT group (*n* = 30), or CN group (*n* = 30). The sample size was calculated considering the expected change of glycosylated hemoglobin (HbA1c) (primary outcome) of the participants. HbA1c is the main parameter to check long-term glycemic control [14]. Previous studies have reported that structured AT or RT had statistically and clinically significant reduction of absolute HbA1c by about 0.6% [21, 22]. Sample size was calculated to have 80% power to detect a moderate 0.65-SD difference in HbA1c, with an alpha/α value of 0.05. Additional participants will be recruited until the required number is achieved (*n* = 38) per group to account for the estimated drop-out rate of 15–25%.

### Randomization and blinding

Adaptive covariate randomization will be carried out to minimize allocation bias and balance important covariates among treatment groups, which has been recommended for clinical research with low sample sizes [23]. Staff who are responsible for exercise administering and data collection will be blinded for the randomization process. Blinding of exercise administers and participants to the intervention is not possible in exercise intervention trials, whereas the outcome assessors (trained staff in independent tertiary care laboratories) will be blinded [22]. Data analysis will be done by the first author on a de-identified database.

### Participant assessment and documentation

An information booklet will be used to record interviewer-administered data collected from participants. Initially, demographic data (name, age, gender, marital status, occupation and income level), screening data for eligibility, and background physical activity levels will be documented. During the study, pre- and post-intervention behavioral, appetite, anthropometry, physical fitness, biochemical parameters and the individual exercise protocols, plus details of each exercise session (24–30 sessions), will be documented by the exercise supervisors.

### Screening

Screening will be undertaken via a thorough medical history and physical activity readiness questionnaire (PAR-Q) [24] followed by a physical examination conducted by a qualified medical practitioner. The objective is to exclude any condition mentioned in the exclusion criteria and ensure safety for progressive exercise. Eligible participants will be allocated to one of the groups and complete a 2D Echocardiogram and Exercise/Stress electrocardiogram/ECG supervised by a consultant cardiologist. Patients who fail the test will be excluded from the study and referred for specialist care (see Fig. 2).

### Intervention

**Fig. 3:**
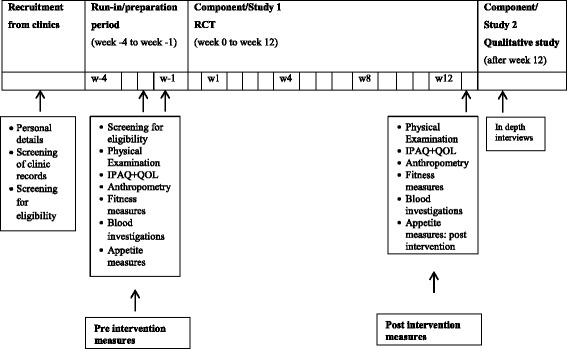
Specific data collection time points in the program

The theoretical basis for the exercise intervention was gleaned from current literature and guidelines from the American College of Sports Medicine (ACSM) and American Diabetes Association (ADA) [25, 26]. Eligible participants will take part in an educational practice period (4 sessions) at the University of Colombo strength gymnasium 2 weeks prior to start of the intervention proper. These sessions will assist participant familiarization with the environment, teach them skills techniques, assess tolerance to the frequency and intensity of exercises etc. Participants will start with minimum tolerable intensity, duration/repetitions and slowly progress to the required exercise dose (AT: 60–70% of HR _max_ for 75 min, RT: 7 exercises all repetitions maintaining 50% repetition maximum (RM) using correct technique). Once participants achieve the minimum requirement, as assessed by the principal investigator, they will start the intervention proper. Pre-intervention (i.e., baseline) parameters will be measured one week before starting the intervention proper (see Fig. 3).

#### RT-intervention group

Participants will follow a supervised progressive resistance training program [26] with each session ~ 60–75 min in duration, 2 times per week for 12 weeks. Duration of each session will be tailored according to the capacity of each individual and progressively accordingly. Exercise duration per week will be approximately 150 min with each session consisting of a warm-up, a resistance training component, and a cool-down phase. There will be a 48-h rest period between each session. With regard to frequency of sessions, a recent review stated there is no significant difference in benefits between 2/week of RT compared to 3/week of RT [27]. The 12-week exercise program will allow adequate time for measurable changes in the group (e.g. muscle fiber hypertrophy typically occurs 6–8 weeks after initiating RT).

The RT phase will target biceps, triceps, pectoralis group, core, quadriceps, hamstring, calf, trapezius, latissimus dorsi and gluteal muscle groups with seven upper body (shoulder press, lateral pull down and biceps curl), lower body (leg press/squat, leg extension, heel lifts,) and core (abdominal crunches starting with pelvic and core muscle activation) exercises. Initial resistance for each exercise will be decided individually by testing strength via 1 repetition maximum/ 1RM [28] and determining maximum weight that can be lifted 8–10 times [17, 29]. The volume of exercise will progress from 1 set of 8 repetitions up to 3 sets (1 × 8 to 3 × 8) and then with increasing resistance according to individual capacity and consistent with the literature [27]. Body resistance, free weights and machines will be used in a circuit manner.

#### AT-intervention group

This group will follow a supervised progressive aerobic training program with exercise intensity progressing up to 60–75% of heart rate max (HR_max_). Heart rate will be measured using Polar heart rate monitors. During the program, brisk walking on the treadmill, stepping up-down, and stationary cycling will be used alternately. Exercise intensity will be progressed according to individual tolerability, graded by HR_max_ and rating of perceived exertion (RPE) using the Borg 6–20 scale. Initially, participants’ workload will be adjusted to achieve 60% of HR_max_ and 8–9/20 RPE for 20–30 min and progressed to 75% of HRmax [29] and 12–13/20 of RPE up to 75 min. Each session will be 75 min in duration, 2 times per week (150 min per week) continuously for 12 weeks with the duration of each session tailored to the capacity of each individual and progressively increased.

#### Control group-active control

This group will be provided with standard care - attending normal clinic visits to a consultant physician prescribing medication and providing general health education. No specific instructions will be provided regarding exercise nor additional information regarding dietary modifications. The group will complete all pre- and post-intervention measurements and participants will be contacted via telephone once every 2 weeks during the 12-week period.

##### Background physical activity

Background physical activity will be assessed in all participants using standard pedometers (Yamax, Digi walker, CW- 600 Japan). Participants will wear a pedometer for 1 full week between weeks 8 and 12 of the intervention, except when showering or sleeping. Background activity is the mean daily total step count for the days the pedometer is worn, excluding steps during scheduled exercise sessions. The International Physical Activity Questionnaire (IPAQ) long version [30] will also be used pre- and post-intervention to assess the physical activity level of participants outside the supervised exercise program. Participants will be advised not to engage in any additional exercise outside the prescribed program during the 3 months.

##### Safety

Participants will be advised about hypoglycemic symptoms during and after the exercise program and about appropriate self-management procedures. Pre- and post-exercise capillary blood glucose will be measured at the initial period and as required to avoid hyper/hypoglycemia during the intervention [31]. Participants with a daily blood glucose level (BGL) exceeding 300 mg/dL or 16.7 mmol/L [17, 26] will not engage in exercise during that day and will be directed to appropriate medical care. BGL less than 100 mg/dL or 5.5 mmol/L [17, 26], will be provided with 15–30 g of glucose and re-assessed by a qualified medical doctor on BGL (< 100 mg/dL) and signs and symptoms of hypoglycemia. They will be allowed to continue/discontinue accordingly. An adequate amount of carbohydrate (glucose) and drinking water will be available to participants during activity to avoid hypoglycemia and dehydration. Blood pressure will be monitored before each session. Emergency drugs and access to hospital will be available in case of an emergency. Special precautions will be taken to prevent injuries and blisters in patients’ feet during the program.

##### Adherence to the intervention

Continuous motivation and advice on adherence to the program will be given during sessions. Standard care in the management of T2DM will be continued and no additional information regarding dietary modifications will be provided. All exercise programs will be prescribed and supervised by a qualified medical doctor (principal investigator). Qualified physiotherapists will be recruited as research assistants to support one-on-one exercise sessions and administration of the study (recruitment of participants, randomization, and completion of questionnaires). Physical training instructors will be available in the gymnasium to provide further support. The principal investigator and exercise supervisors will be trained in clinical exercise prescription.

### Intervention fidelity

Three exercise supervisors will deliver the exercise protocol in a uniform. At random time points, an independent assessor will observe classes and monitor content consistency using a checklist on explicit areas of the exercise protocol. The principal investigator will check the exercise protocol log daily and the independent assessor will review it at random intervals to check that the intervention is delivered as intended.

### Outcome measures

All outcomes are measured pre- (within one week before the start date of the intervention) and post-intervention (within one week after the intervention) (Table 1).

**Table 1:** Outcome variables and measuring instruments/procedures

Outcome variable	Measuring instrument/procedure
Anthropometry: Height, weight, body circumferences (waist, hip, mid-arm, mid-thigh)	∙ISAK guidelines. ∙Stadiometer, electronic weighing scale and measuring tape (SECA, Germany)
Body fat %	∙Seven site skin fold measures by ISAK and ACSM guidelines. ∙Harpenden skin fold caliper.
Total and regional body composition	∙Dual energy X- ray absorptiometry (DXA) full body scan. Hologic Discovery W, DXA system.
Cardiovascular endurance/Fitness	∙6 min walk test (6MWT). ∙Queens College/McArdle Step test. ∙Exercise Electrocardiogram (ECG).
Muscle strength	∙ACSM guidelines. ∙One repetition maximum (1RM) testing with machine and free weights (Precor, USA).
Blood pressure	OMRON digital blood pressure monitor (Japan).
Heart rate	Polar heart rate monitors.
Biochemical parameters: Glycemic control (HbA1c FBS, Fasting insulin), lipid level, liver enzymes, HsCRP.	Venous blood samples with overnight fast tested in tertiary care independent laboratory.
Quality of life	SF-36 quality-of-life outcomes questionnaire. Self-administered.
Liking and wanting for different types of food	Computer-based behavioral procedure Leeds Food Preference Questionnaire (LFPQ).Self-administered.

### Primary outcome

#### Change in glycemic control

Primary outcome will be absolute change in glycosylated hemoglobin (HbA1c) levels. HbA1c is the accepted parameter to monitor long-term glycemic control (normal values are 4–6.5%) [32]. Additionally, fasting blood sugar levels (FBS) and fasting insulin (FI) levels will be measured to further assess and compare change in insulin resistance status. A venous blood sample will be collected after an overnight fast by a trained medical officer/investigator and transported to a standard tertiary care laboratory for analysis. Laboratory technicians are blinded to the study participants.

### Secondary outcomes

#### Change in percentage body fat (%BF)

Total body fat percentage will be measured via dual energy X-ray absorptiometry (DXA) full body scan [33], a gold standard method for the assessment of body composition. Additional details on regional body fat will be provided. The DXA scan is completed in a standard tertiary care laboratory by a trained technician who is blinded to the study participants. Body composition will also be assessed using seven skin fold thickness measures (triceps, chest, sub scapular, axillary, mid-thigh, abdominal and suprailiac skin folds) [31] with a Harpenden skin fold caliper by the trained principal investigator. All measurements will be taken in the morning between 0900 and 1000 am by the same investigator to maintain consistency and inter-rater reliability. The ISAK (International Society for the Advancement of Kinanthropometry) methodology and American College of Sports Medicine Guidelines will be used [34].

Skinfold thickness, body circumferences, and DXA will enable a detailed appraisal of total and regional adiposity. The addition of waist circumference and skinfold thickness measures to DXA values will improve data on central adiposity [33].

### Lipid profile, liver profile and chronic inflammatory status

- Absolute change in plasma lipid profile, total cholesterol (TC), triglycerides (TG), high-density lipoproteins (HDLC), low-density lipoproteins (LDLC) will indicate change in metabolic and cardiovascular risk of participants [35].
- Change in highly sensitive serum C-reactive protein (hs-CRP) will be measured to identify the chronic inflammatory status, a predictor of future cardiovascular risk [36].
- Serum Aspartate aminotransferase/Alanine aminotransferase (AST/ALT) levels will be measured to assess liver cell damage consistent with metabolic derangement in metabolic syndrome and T2DM [37].

### Anthropometry and cardiovascular/fitness parameters

- Anthropometry:

*Measurements* will be taken in the morning between 0900 and 1000 am adhering to ISAK guidelines by the trained principal investigator who is a qualified medical practitioner. Height will be measured while the participant is barefoot to the nearest 0.1 cm using a portable stadiometer (Seca 213, Germany). Weight will be measured (wearing light clothing without shoes) using an electronic weighing scale (Seca 813, Germany) and recorded to the nearest 0.1 kg. Body Mass Index (BMI) will be calculated using the standard calculation (weight/height ^2^ kgm^− 2^). *Waist, hip, mid-arm, mid-thigh circumferences* will be measured using a non-elastic measuring tape (Seca 201, Germany) to the nearest 0.1 cm. A mean value will be recorded after three consecutive measurements [31].

- Cardiovascular endurance and electrocardiographic measures:

1. Exercise ECG will be undertaken at a tertiary care hospital electrocardiographic laboratory under the supervision of a consultant cardiologist and trained staff using the Bruce protocol. Exercise ECG results will provide profile electrocardiographic changes in addition to blood pressure and heart rate changes to progressive exercise.
2. YMCA 3-Minute Step Test will be used to measure heart rate changes with acute progression of exercise [31].
3. The 6-min walk test (6MWT) will be used to measure endurance and the walkability of participants [31].

- Resting and exercise heart rate will be monitored by heart rate monitors (Polar). Systolic and diastolic blood pressure will be measured using standard methods by trained research assistants using an Omron HEM-7130 electronic sphygmomanometer.
- Muscular strength: One Repetition Maximum (1RM) will be assessed in all major muscle groups [31].

### Quality of life (QoL)

Change of Quality of Life will be assessed using the SF-36 quality-of-life outcomes questionnaire [38] previously validated in Sri Lanka.

### Liking and wanting of food

Change in preference for a particular type of food (high fat: sweet, high fat: savory, low fat: sweet, low fat savory) will be assessed using the computer-based Leeds Food Preference Questionnaire (LFPQ) [39]. This questionnaire will be customized to the Sri Lankan population by adding Sri Lankan foods to different food categories asked in the questions. The participant will initially be trained to use the computer program by the investigator before being self-administered.

### Qualitative study

The primary outcome of the qualitative study is to identify barriers and facilitators experienced during the prescribed exercise programs using in-depth interviews (IDIs). This will be carried out after the last exercise session of the individual program by the principal investigator and a trained assistant. Face-to-face interviews will be conducted with privacy maintained using a set of pre-determined semi-structured open-ended questions regarding; the stage of behavioral change, knowledge of exercise, individual’s beliefs, perceived ability, limitations/barriers and facilitators experienced during the prescribed exercise programs.

During interviews, written notes will be taken and responses from participants will be audio recorded, including emotional responses. The number of interviews will determined by data saturation which will be revealed during concurrent analysis.

To the best of our knowledge, no studies have been conducted to date where the effectiveness of a RCT has been assessed via the qualitative experience of participants. This component will allow a triangulation approach to incorporate quantitative and qualitative data at the stage of results interpretation.

### Data and statistical analysis

Quantitative data will be processed and analyzed using SPSS version 20. To measure pre- and post-intervention absolute changes in glycaemia, anthropometry, body composition, metabolic and fitness parameters, liking/wanting for food and quality of life across the groups; a linear mixed model analysis for repeated measures that includes the covariates age and sex will be used. The SF-36 will be scored according to the guidelines provided by the RAND Corporation [40] and additionally effect size (Cohen’s d) will be assessed to determine change effect of each scale. The LFPQ will use Visual Analogue Scale (VAS) scores for explicit liking/ wanting for food and standardized ‘d-score’ (D-RT) for implicit wanting for food using E-prime software. The effect *p* = 0.05 will be considered statistically significant.

Qualitative data of the interviews will be audio recorded and verbatim transcribed for analysis. The facilitator and two observers will analyze their respective IDI using their notes (verbal and non-verbal responses of participants) and the tape-recorded data collectively immediately or within 24 h after the conclusion of the IDI. Transcripts will then be downloaded, coded, and analyzed for emergent themes using NVivo 10 qualitative analysis software [41]. The differences in coding will be resolved via discussions by a group of independent reviewers.

The data will be stored for future use after the completion of the study.

## Discussion

Findings from the proposed study are expected to benefit the knowledge base of health professionals in the South Asian region and provide further evidence for the development of specialised clinical exercise physiology services in the country.

Despite the high prevalence of T2DM in Sri Lanka, there are no published data on the effects of exercise in patients with the condition. Further, no adequately powered methodologically advanced trials have been conducted in South Asia to quantify multiple parameters regarding different modes of exercise prescription for patients with diabetes.

Differences in the body composition and metabolic profile of South Asians may differentially influence the relative effects of aerobic and resistance exercises, compared to other ethnicities. Further, the effects of exercise on food preference has not been investigated among South Asians and results will likely allow for better planning of lifestyle interventions for Sri Lankans.

Globally, the management of chronic non-communicable diseases (NCDs) is changing from a ‘disease’ to a ‘health’ model with a greater focus on lifestyle modification. This study is planned in a society where recreational ‘exercise culture’ is not a norm and the development of the new processes and services will be challenging. Findings of the proposed study will inform the development of a much needed professionally driven specialized service in behavior change and lifestyle modification to combat the NCD epidemic in the region.

The addition of a qualitative study to assess the barriers and facilitators for adherence to the intervention is also novel. Finally, we plan to triangulate quantitative and qualitative outcomes and propose a culturally adaptable therapeutic physical activity intervention to Sri Lankan patients with T2DM and inform policy makers.

## Abbreviations

ACSM: American College of Sports Medicine
AT: Supervised progressive aerobic exercise program
BGL: Blood glucose level
CN: Control group
ECG: Electrocardiogram
FBS: Fasting blood sugar
HbA1c: Glycosylated hemoglobin
HRmax: Maximum Heart Rate
NCD: Non Communicable Diseases
RCT: Randomized controlled trial
RT: Supervised progressive resistance exercise program
SA: South Asians
T2DM: Type 2 diabetes mellitus
